# Supplementary figures and images for: Postharvest biochemical characteristics and ultrastructure of Coprinus comatus
Source: PeerJ. 2020 Feb 5;8:e8508. doi: 10.7717/peerj.8508 (PMC7007737; doi:10.7717/peerj.8508)

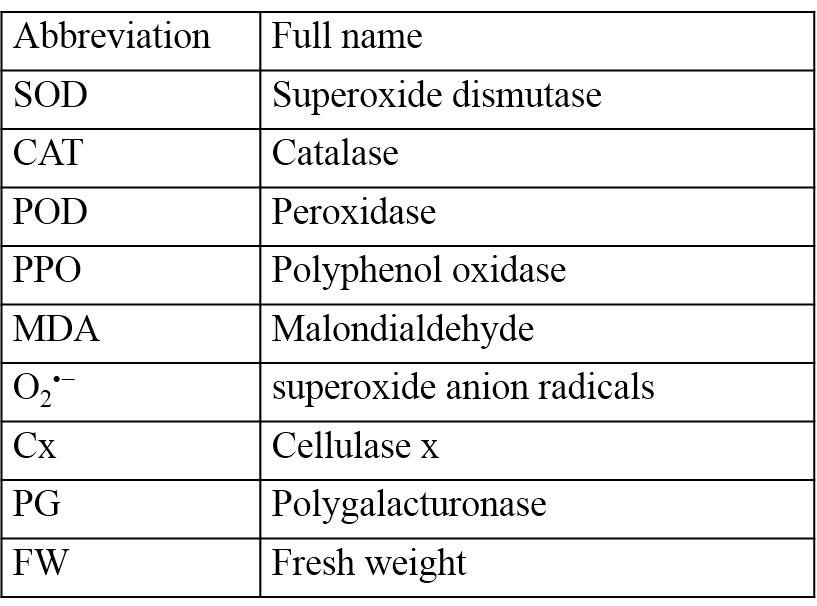

Supplement: Supplemental Information 1 [file peerj-08-8508-s001.png]

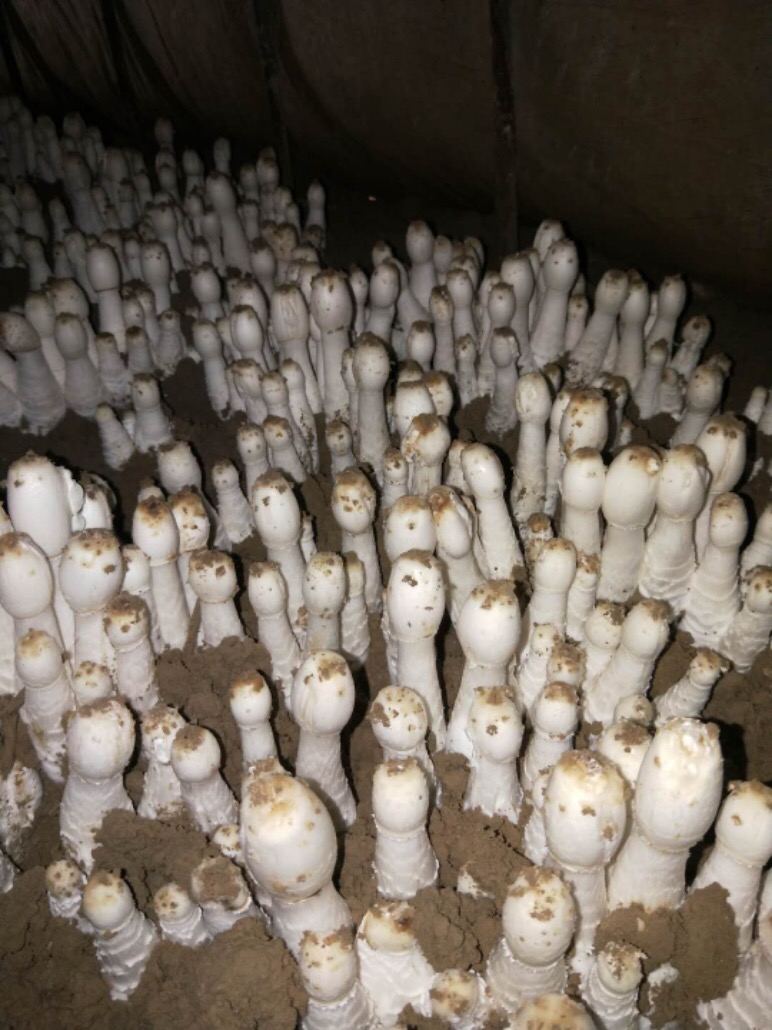

Supplement: Supplemental Information 2 [file peerj-08-8508-s002.jpg]
